# Gene-gene interaction filtering with ensemble of filters

**DOI:** 10.1186/1471-2105-12-S1-S10

**Published:** 2011-02-15

**Authors:** Pengyi Yang, Joshua WK Ho, Yee Hwa Yang, Bing B Zhou

**Affiliations:** 1 School of Information Technologies, University of Sydney, NSW 2006, Australia; 2 School of Mathematics and Statistics, University of Sydney, NSW 2006, Australia; 3 National ICT Australia, Australian Technology Park, Eveleigh, NSW 2015, Australia; 4 Centre for Distributed and High Performance Computing, University of Sydney, NSW 2006, Australia

## Abstract

**Background:**

Complex diseases are commonly caused by multiple genes and their interactions with each other. Genome-wide association (GWA) studies provide us the opportunity to capture those disease associated genes and gene-gene interactions through panels of SNP markers. However, a proper filtering procedure is critical to reduce the search space prior to the computationally intensive gene-gene interaction identification step. In this study, we show that two commonly used SNP-SNP interaction filtering algorithms, ReliefF and tuned ReliefF (TuRF), are sensitive to the order of the samples in the dataset, giving rise to unstable and suboptimal results. However, we observe that the ‘unstable’ results from multiple runs of these algorithms can provide valuable information about the dataset. We therefore hypothesize that aggregating results from multiple runs of the algorithm may improve the filtering performance.

**Results:**

We propose a simple and effective ensemble approach in which the results from multiple runs of an unstable filter are aggregated based on the general theory of ensemble learning. The ensemble versions of the ReliefF and TuRF algorithms, referred to as ReliefF-E and TuRF-E, are robust to sample order dependency and enable a more informative investigation of data characteristics. Using simulated and real datasets, we demonstrate that both the ensemble of ReliefF and the ensemble of TuRF can generate a much more stable SNP ranking than the original algorithms. Furthermore, the ensemble of TuRF achieved the highest success rate in comparison to many state-of-the-art algorithms as well as traditional *χ*^2^-test and odds ratio methods in terms of retaining gene-gene interactions.

## Background

The advancement of high-throughput genome-wide association (GWA) studies has tremendously improved our understanding of the genetic basis of many common complex diseases [1]. Under the assumption that common diseases are associated with common variants, GWA studies often aim to identify a set of single nucleotide polymorphisms (SNPs) that are statistically associated with a target disease. Typically, this is achieved by adopting a case-control study design that perspectively identify genotypes (SNP combinations) that distinguish individuals who have a certain disease (case) from a control population of individuals (control) [2].

Several recent studies indicate that many complex traits cannot be explained by any single SNP variants and the characterization of gene-gene interactions and gene-environment interactions may be the key to understand the underlying pathogenesis of these complex diseases [3-5]. For this reason, several methods have been developed to jointly evaluate SNP and environmental factors with the aim to identify gene-gene and gene-environment interactions that have major contributions to complex diseases [6]. These methods analyze genetic factors in a combinatorial manner when applied to SNP dataset with case and control samples. Therefore, we shall refer to them as *combinatorial methods*. Popular combinatorial methods include *random forests* based algorithms [7,8], multifactor dimensionality reduction (MDR) [9,10], Bayesian based algorithms [11], and evolutionary approaches [12,13].

Combinatorial methods are computationally intensive and the computation time increases exponentially with the number of SNPs considered. Therefore, it is of great interest to perform a filtering step prior to the combinatorial evaluation to remove as many irrelevant SNPs as possible [14]. This is commonly known as the two-step analysis approach [3]. As discussed in a number of recent reviews [3,4,15], a good filtering algorithm is of critical importance since if the functional SNPs are removed by the filter, the subsequent combinatorial analysis will be in vain.

For categorical data such as genotypes of SNPs, univariate filtering algorithms including *χ*^2^-test and *odds ratio* are commonly used. However, these methods consider the association between each SNP and the class label independently of other SNPs in the dataset [16]. Therefore they may filter out SNP pairs that have strong interaction effects but have weak individual association with the phenotype [4]. Recently, new multivariate approaches known as “ReliefF-based” filtering algorithms were proposed. This series of new methods, including ReliefF [17], tuned ReliefF (TuRF) [18], and Spatially Uniform ReliefF (SURF) [19] takes into account dependencies between attributes [17]. This is critical for preserving and prioritizing potential gene-gene interactions in SNP filtering [20].

Although ReliefF-based methods have gained much attention and have been applied to several association studies (*e.g.*, [21]; and [22]), we found that filtering results produced by ReliefF and TuRF are sensitive to the order of samples presented in the dataset. By investigating the ReliefF algorithm, we identify that such a sample order dependency is related to an intrinsic tie-breaking procedure inherited in the *k*-nearest neighbors (*k*NN) routine. It causes a partial utilization of neighbor information, leading ReliefF and TuRF to generate unstable results. While such an unstable behavior appears to be undesirable, it is an important characteristic for ensemble learning [23].

In this study, we propose an ensemble approach to obtain a more faithful survey of the set of nearest neighbors to each target sample. This is accomplished by aggregating the ranking score generated from multiple filters on datasets with permutated sample order. The proposed ensemble approach extends the idea of a classification-oriented ensemble feature selection method [24] which uses a bootstrap sampling procedure with multiple filters to produce different rankings. However, the proposed ensemble approach is more powerful because the entire dataset (in contrast to a bootstrap subset) is used for ensemble learning.

Using simulated and real SNP datasets, we demonstrate that the proposed approach not only can generate much more stable SNP ranking results, the ensemble of TuRF can vastly improve the success rate of retaining functional SNP pairs compared to many other traditional as well as state-of-the-art SNP filtering methods.

## Methods

Consider a GWA study consisting of *N* SNPs and *M* samples. Let us define each SNP in the study as *g_j_* and each sample as *s_i_* where *j* = 1*...N* and *i* = 1*...M*. The aim of the filtering procedure is to produce a ranking score defined as *W*(*g_j_*) and commonly refers to as weight. This score represents the ability of each SNP *g_j_* to separate samples between the case and control groups, and the filtering is done by removing those with low ranking scores according to a pre-defined threshold.

### Existing ReliefF-based algorithms

In ReliefF algorithm, the weight score of each SNP, *W*(*g_j_*), is updated at each iteration as follows [25]:

*W*(*g_j_*) *= W*(*g_j_*) *– D*(*g_j_,s_i_,h_k_*)*/M + D*(*g_j_,s_i_,m_k_*)*/M* (1)

where *s_i_* is the *i^th^* sample from the dataset and *h_k_* is the *k^th^* nearest neighbor of *s* with same class label (called “hit”) while *m_k_* is the *k^th^* nearest neighbor to *s_i_* with different class label (called “miss”). This weight updating process is repeated for *M* samples selected randomly or exhaustively. Therefore, dividing by *M* keeps the value of *W*(*g_j_*) to be in the interval [-1, 1]. *D*(*.*) is the difference function that calculates the difference between any two samples *s_a_* and *s_b_* for a given gene *g*:(2)

where *G*(*.*) denotes the genotype of SNP *g* for sample *s*. The nearest neighbors to a sample are determined by the distance function, *MD*(*.*), between the pairs of samples (denoted as *s_a_* and *s_b_*) which is also based on the difference function (Eq. 2):(3)

Using pseudocode, we can outline the ReliefF algorithm in **Algorithm 1.**

The ReliefF algorithm calculates the distance between different samples using the genotype information of all SNPs. However, such a procedure is sensitive to noise in the dataset.

**Algorithm 1** ReliefF

1: **for***j =* 1 to *N***do**

2: initiate(*W*(*g_j_*));

3: **end for**

4: **for***i =* 1 to *M***do**

5: *s_i_ =* randomSelect(*sampleSize*);

6: *H =* findHitNeighbours(*s_i_,K*); (*h*_1_...*h_K_ ∈ H* )

7: *M =* findMissNeighbours(*s_i_,K*); (*m*_1_...*m_K_ ∈ M*)

8: **for***j =* 1 to *N***do**

9: **for***k =* 1 to *K***do**

10: *W*(*g_j_*) *= W*(*g_j_*) *– D*(*g_j_, s_i_, h_k_*)*/M + D*(*g_j_, s_i_, m_k_*)*/M*

11: **end for**

12: **end for**

13: **end for**

TuRF [18] aims to improve the performance of the ReliefF algorithm in SNP filtering by adding an iterative component. The signal-to-noise ratio is enhanced significantly by recursively removing the low-ranked SNPs in each iteration. Specifically, if the number of iteration of this algorithm is set to *R,* it removes the *N/R* lowest ranking (*i.e.,* least discriminative) SNPs in each iteration, where *N* is the total number of SNPs. The pseudocode for TuRF is shown in **Algorithm 2.**

**Algorithm 2** TuRF

1: **for***i =* 1 to *R***do**

2: apply ReliefF(*M*,*K*);

3: sortSNP();

4: removeLowSNP(*N / R*);

5: **end for**

6: **return** last ReliefF estimate for each SNP

We follow the same configuration in previous studies [18,19,25], in which exhaustive sample selection (*i.e., M* is set to be the number of training instance, and the order of sample to be evaluated is the same as the order presented in the dataset) is adopted, *K =* 10 of nearest neighbors is used, and 10 iterations (*R =* 10) for TuRF is applied.

### Ensemble of ReliefF and TuRF

We find that the ReliefF algorithm is sensitive to the order of samples used to calculate the SNP ranking score (Eq. 1). That is, running these algorithms on the same dataset in which the order of the samples is permuted (while maintaining the sample-class label association), leads to different SNP ranking results.

Such a sample order dependency is related to the assignment of “hit” and “miss” nearest neighbors of each sample (lines 6 and 7 of **Algorithm 1**). Since *K* nearest neighbors are calculated by comparing the distance between each sample in the dataset (using all the SNP attributes) and the target sample (*s_i_* in **Algorithm 1**), a tie occurs when more than *K* samples have a distance equal or less than the *K^th^* nearest neighbor of *s_i_*. We can show that the sample order dependency can be caused by using any tie breaking procedure which forces exactly *K* samples out of all possible candidates to be the nearest neighbors of *s_i_*, which causes a different assignment of “hit” and “miss” of nearest neighbors when the sample order is permuted.

Aiming to increase the stability and the power of SNP-SNP interaction filtering, here we propose an algorithm that (1) preserves the general algorithmic principle of ReliefF, and (2) make use of all the information embedded in all the tied samples when a tie-breaking situation occurs. To achieve this, we use a rank score aggregation approach that adhere to the general principle of ensemble learning [23]. From our analysis of the tie-breaking problem aforementioned, it is clear that a different set of samples may be assigned to be a sample’s nearest neighbors. Therefore, the result of a single run of ReliefF utilizes only partial information embedded in the full set of the nearest neighbors. In order words, the results from multiple runs of ReliefF using the dataset with permuted sample order should contains complementary information about how well each set of SNPs can discriminate between the two classes (case vs. control). In this sense, we can potentially harness the “diversity” [26] of ranking results from multiple execution with permuted sample order using an ensemble-based methods to produce more stable and accurate SNP ranking results.

Formally, our ensemble of ReliefF (called ReliefF-E) produces *L* copies of the input SNP dataset D*_l_* (*l =* 1, ...,*L*) by randomly permuting the order of the samples. ReliefF-E calculates the weight for each SNP *g_j_* by averaging multiple rankings *rank*(*h_l_*(*g_j_*), D*_l_*) each from a permuted dataset as follows:(4)

where *W_ensemble_*(*.*) is the ensemble weight and *h_l_*(*.*) is the *hypothesis* of a filter algorithm obtained from the permuted dataset D*_l_*.

Similarly, the ensemble of TuRF (called TuRF-E) performs multiple runs of TuRF, and aggregates the ranking scores of each SNP produced in each iteration of TuRF using Eq. 4.

### Analysis of simulated and real-world datasets

We performed a set of analysis on simulated and real-world SNP datasets to (1) demonstrate the sample order dependency in ReliefF and TuRF, (2) show that ReliefF-E and TuRF-E can indeed largely eliminate such a sample order dependency, and (3) investigate whether our ensemble approaches can effectively retain functional SNP pairs. We used a set of simulated datasets generated by [27] in our analysis. These simulation datasets were generated using different genetic models (different heritability and sample size) and each model randomly simulated the genotype of 1000 SNPs across all the samples except for one functional SNP-SNP interaction pair denoted as “X0” and “X1” in the dataset. These datasets are summarized in Table 1.

**Table 1:** Summary of simulation datasets. Each model contains 100 datasets.

Model	SNP size	Sample size	Heritability
Epistatic_400_0.05	1000	case: 200; control: 200	0.05
Epistatic_400_0.1	1000	case: 200; control: 200	0.1
Epistatic_400_0.2	1000	case: 200; control: 200	0.2
Epistatic_400_0.3	1000	case: 200; control: 200	0.3

Epistatic_800_0.05	1000	case: 400; control: 400	0.05
Epistatic_800_0.1	1000	case: 400; control: 400	0.1
Epistatic_800_0.2	1000	case: 400; control: 400	0.2
Epistatic_800_0.3	1000	case: 400; control: 400	0.3

A GWA study dataset generated from case-control design of age-related macular degeneration (AMD) samples [28] is also used to illustrate the sample order dependency of ReliefF and TuRF when applied to real SNP datasets. The AMD dataset contains 96 cases and 50 controls, with the genotype of 116,212 SNPs for each sample.

To demonstrate the sample order dependency, a dataset is analyzed by each of the four filtering algorithms (ReliefF, TuRF, ReliefF-E, and TuRF-E) twice in which a different permutation of sample order is used for each run of the algorithm (yet we note that similar results are consistently obtained in all repeated experiments with different permutation of sample order). A Pearson correlation coefficient, *r*, is calculated using the rank of all SNPs generated from the two runs. A SNP filtering algorithm that obey sample order invariance should produce the same SNP ranking regardless of the sample order (*i.e.*, *r =* 1).

These simulated datasets (Table 1) are also used to investigate how well each algorithm can retain functional SNP pair while performing SNP filtering. Methods included in this comparison are traditional filters: *χ*^2^-test and odds ratio; ReliefF-based filters: ReliefF, TuRF, and SURFTuRF; and two ensemble filters: ReliefF-E and TuRF-E. Specifically, we apply these seven filtering algorithms to the eight simulated models and compare the success rate of each method in filtering 100 datasets of each model. The success rate is defined as the number of times a give filtering algorithm is able to retain the interaction SNP pair in the dimension reduced subset in 100 datasets. The dimension of the dataset is divided in percentile. For a dataset with a SNP size of 1000, the percentile of 1 includes the top 10 SNPs while the percentile of 10 includes the top 100 SNPs. Therefore, if we reduce the dimension of the dataset to 100 SNPs (that is, the percentile of 10), and the interaction SNP pair is within this 100 SNPs, we say the filter successfully retained the interaction SNP pair at the percentile of 10.

We use graph to present the success rate of each method from 1 to 50 percentile, and we quantify the overall success rate of each method by an *average cumulative success rate* which is computed as the sum of success rate from percentile 1 to 50 divided by 50.

## Results

### The effect of the sample order dependency

**Figure 1:**
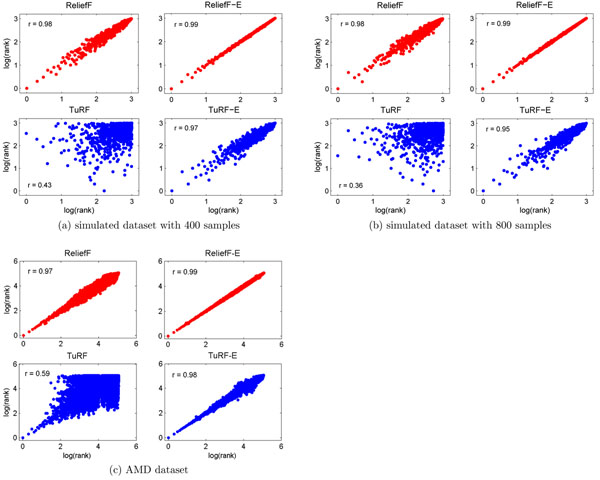
**Correlation comparison.** The correlation between SNP ranking (log_10_ transformed) generated by two runs of ReliefF, TuRF, ReliefF-E, and TuRF-E using a simulated datasets (400 and 800 samples) and the AMD dataset in which each run use a different sample order.

We found that the SNP ranking generated by ReliefF and TuRF are sensitive to the order in which the samples are presented in the dataset. Figure 1(a) shows the Pearson correlation of the ranking of the SNPs in two separate runs of ReliefF and TuRF using a dataset containing 1000 SNPs and 400 samples (200 controls and 200 cases). Figure 1(b) is the result of the same analysis applied to a simulated dataset containing 800 samples. It is clear that both ReliefF and TuRF algorithms are sensitive to the order of samples presented in datasets, causing the rank of each SNP inconsistent between the original dataset and the randomly re-ordered dataset. While such an inconsistency is relatively small for the ReliefF algorithm, the problem is much more severe in TuRF. The Pearson’s correlation coefficient of two runs of TuRF is *r =* 0.43 for the dataset with 400 samples and a *r =* 0.36 for the dataset with 800 samples. As we shall demonstrate later, such an instability caused by sample order dependency has led TuRF to perform suboptimally.

By using the ensemble approach (an ensemble size of 50; see Section “Determining ensemble size” for details), we are able to stabilize the ranking results of both ReliefF and TuRF. Especially, TuRF-E can significantly increase the stability of the SNP ranking results of TuRF, with a *r =* 0.97 for the dataset with 400 samples and a *r =* 0.95 for the dataset with 800 samples.

Similar results were obtained when the AMD dataset was analyzed (Figure 1(c)). The results illustrate that the sample order instability is indeed a problem in analyzing real biological datasets with ReliefF and TuRF. The use of our ensemble approach increases stability and this is evident from the increase of the ranking correlation to a *r =* 0.99 for ReliefF and a *r =* 0.98 for TuRF.

### The origin of the sample order dependency

**Figure 2:**
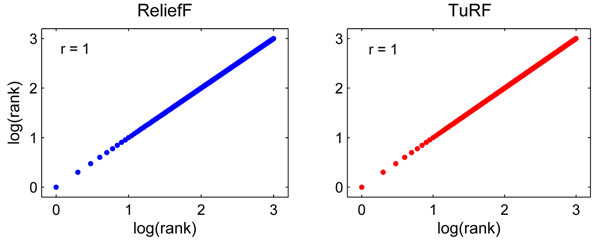
**Correlation with tie causing samples removed.** The correlation between the SNP ranks (log_10_ transformed) of two separate runs using datasets with tie causing samples removed.

To verify whether the sample order dependency is indeed caused by tie breaking, we modified and recompiled the source code of mdr-2.0_beta_6.zip (downloaded from http://sourceforge.net/projects/mdr/) to report the tie causing samples and remove them from the dataset. After removing all tie causing samples, we were able to obtain completely reproducible ranking results (*i.e.*, *r =* 1) with both ReliefF and TuRF (Figure 2). However, resolve sample order dependency using this approach requires aggressive removal of a large number of samples, which inevitably reduces the algorithms’ power to filter functional SNP pairs.

One tempting way to solve such a sample order dependency is to use a randomize procedure to select a sample randomly when a tie occur. However, our experiments indicate that such a procedure does not increase the correlation (data not shown). In fact, any tie-breaking procedure which chooses one sample out of all valid candidate samples will necessarily produce instability in its resulting ranking score.

Another way to solve such a sample order dependency can be achieved by defining nearest neighbors to a sample as the ones that are within a certain distance threshold of the target sample. A recently developed variant algorithm of ReliefF called SURF (Spatially Uniform ReliefF) [19] employed this idea. However, by doing so, the algorithm will rely directly on a predefined threshold for nearest neighbors selection, which may negatively affect the result giving the sample sparsity in high-dimensional space. Therefore, such an approach lack the robustness of the rank based *k*NN criteria. Indeed, our evaluation (which is presented in later section) confirmed that SURF does not fully recover the SNP filtering capability. As discussed later in this paper, our ensemble approach, which rely on sample ranking instead of direct thresholding, gives consistently better results.

### Determining ensemble size

**Figure 3:**
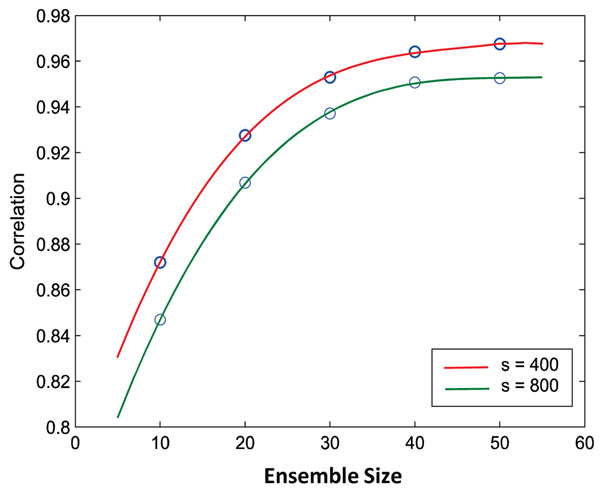
**Ensemble size determination.** The correlation between the SNP ranks with respect to different ensemble sizes of TuRF using simulated datasets with 400 samples (s=400) and 800 samples (s=800).

An important parameter in any ensemble method is the ensemble size. In our case, it is the number of times an algorithm is repeatedly applied on a dataset with reordered samples. It is important to estimate the minimum ensemble size that is sufficient to reduce sample order dependency. We estimate this value via repeating the correlation analysis on TuRF-E with an ensemble size of 10, 20, 30, 40, and 50 using the simulated datasets with 400 samples and 800 samples (Figure 3). It is apparent that the increase of the correlation in two separate runs using the original and the randomly re-ordered datasets plateaus at around an ensemble size of 40 for both datasets, and there is only minor improvement when employing more than 50 runs. Therefore, the ensemble size of 50 is used in all our experiments in this study.

### Ensemble approach to improve retention rate of functional SNP pairs in SNP filtering

**Figure 4:**
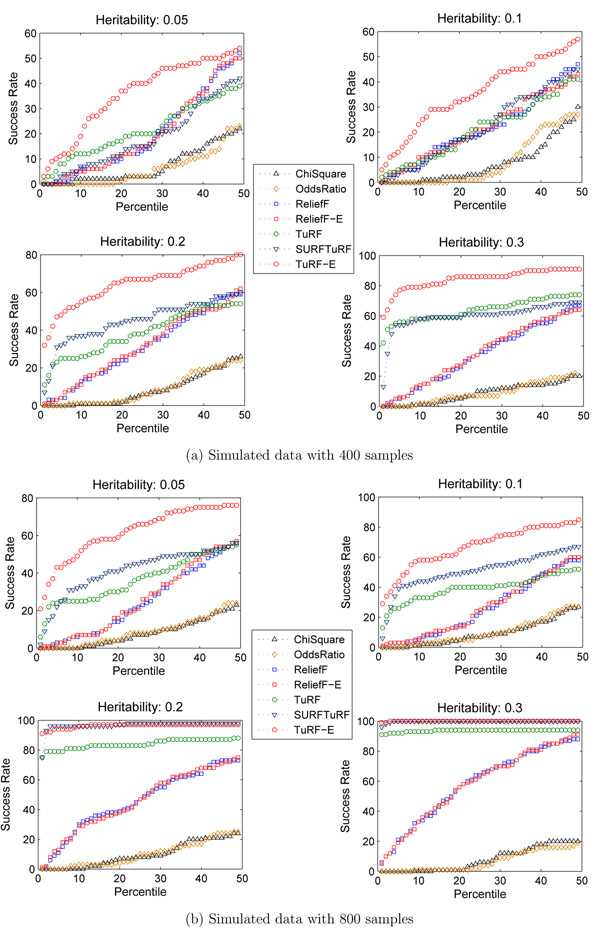
**Success rate comparison using different filtering algorithms.** The comparison of success rate for retaining the functional SNP pair in simulated datasets using 7 different filtering algorithms.

One motivation of using the proposed ensemble approach is to gain a more informative SNP scoring. Therefore, we investigated whether our ensemble scheme can improve the ability of ReliefF and TuRF to retain functional SNP pairs in SNP filtering. Figure 4 shows the trend of the success rate of each filtering algorithms across percentile 1 to 50 (*i.e.*, 10-500 top ranking SNPs) using simulated datasets with 400 sample and 800 sample respectively. We then repeated ReliefF and its corresponding ReliefF-E, TuRF and its corresponding TuRF-E each five times to calculate the standard deviation in multiple runs. Table 2 shows the average cumulative success rate of these algorithms on the same set of simulated datasets. We found that TuRF-E performed the best in all cases examined in this study regardless of sample size and heritability of the simulated datasets. ReliefF-E and ReliefF have similar performance in terms of success rate, while traditional univariate filters such as *χ*^2^-test and odds ratio give the lowest success rates. The superiority of TuRF-E is particularly noticeable in datasets simulated with low heritability or small number of samples. This implies that TuRF-E is applicable in even these “challenging” cases where other ReliefF-based algorithms fails to achieve high enough success rates.

**Table 2:** Average cumulative success rate from percentile 1 to 50 using the simulated datasets (400 and 800 samples). The best algorithm with the highest average cumulative success rate in each dataset is shown in bold.

Methods	Heritability = 0.05	Heritability = 0.1	Heritability = 0.2	Heritability = 0.3
Simulated dataset with 400 samples				
χ^2^-test	6.92	7.20	8.06	8.51
Odds Ratio	5.86	7.84	8.43	8.58
ReliefF	18.96±0.38	20.93±0.47	30.35±0.28	33.98±0.31
ReliefF-E	19.27±0.17	21.22±0.14	30.92±0.24	34.76±0.26
TuRF	22.11±1.34	24.59±2.53	42.27±3.41	61.37±1.58
SURFTuRF	18.12	21.88	44.92	59.88
TuRF-E	**35.23±0.37**	**35.85±0.82**	**63.55±0.93**	**84.71±0.25**

Simulated dataset with 800 samples				
χ^2^-test	7.73	8.53	9.61	7.84
Odds Ratio	8.53	9.86	9.92	6.61
ReliefF	24.37±0.52	25.11±0.80	44.23±0.86	54.40±0.75
ReliefF-E	25.59±0.63	25.85±0.28	44.81±0.36	56.91±0.46
TuRF	33.20±2.11	39.99±2.04	78.64±3.14	91.93±1.13
SURFTuRF	41.20	50.82	96.27	99.86
TuRF-E	**61.59±0.58**	**65.75±1.09**	**96.69±0.26**	**99.96±0.21**

ReliefF-E only has marginal improvement over ReliefF whereas TuRF-E achieves a significant improvement over TuRF. This is likely due to the fact that the TuRF algorithm executes ReliefF multiple times while removing low ranking SNPs in each iteration. Therefore, the ensemble approach could accumulate more information in each iteration. It is also observed that SURFTuRF does not improve on TuRF in analyzing datasets of 400 samples. This is consistent with our hypothesis that a predefined distance threshold in SURFTuRF may be sensitive to high SNP-to-sample ratio (thus, high-dimensionality). Moreover, The standard deviations of ReliefF and TuRF are generally much larger than their ensemble version, indicating the sample order dependency also affecting the stability of the success rate of SNP-SNP filtering.

**Figure 5:**
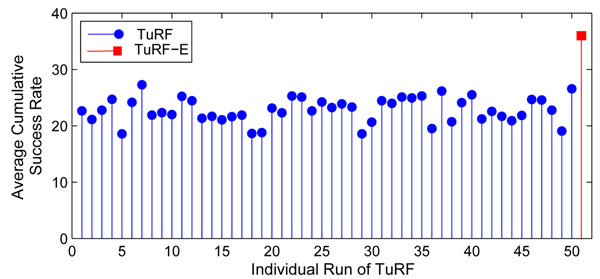
**Average cumulative success rate comparison of TuRF-E and multiple TuRF runs**. Comparison of average cumulative success rate of 50 individual runs of TuRF (shown in a blue circle) and their aggregate results (TuRF-E; shown in a red square) using a simulated dataset with 400 samples (heritability = 0.05).

We further investigated whether TuRF-E is simply “averaging” out the detection ability at different runs of TuRF. Figure 5 shows the average cumulative success rates of 50 runs of TuRF on a simulated dataset (sample size = 400, heritability = 0.05) where a different sample order is used in each run, and the corresponding average cumulative success rate of their aggregate version (TuRF-E). It is clear that the aggregate SNP ranking result is significantly better than any single run of TuRF. This implies that our aggregation algorithm is indeed able to make use of the information embedded in multiple runs of TuRF to improve its detection ability, verifying our motivation of using an aggregation approach.

## Discussion

The field of gene-gene and gene-environment interaction identification from GWAS data is still young and rapidly developing. One of the biggest challenges in identification of such interaction relationship is computational efficiency since in the worst case an exponentially large number of SNP combinations need to be evaluated. As discussed by a number of authors [3,4,15], effective SNP filtering can greatly reduce the computational burden of the subsequent combinatorial evaluation by removing a large portion of noise. The main advantage of using ReliefF based algorithms for SNP filtering is that they can detect conditional dependencies between attributes [17]. Furthermore, they are computationally efficient. A good implementation of TuRF can analyze a GWA study data with up a few hundred samples in the order of minutes. Such computationally efficiency, coupled with its intrinsic ability in detecting SNP dependencies, has led to its increasing wide-spread applications.

Through analyzing the ReliefF-based algorithms, we discovered a previously unknown anomaly in both ReliefF and TuRF. We show these two popular filtering algorithms are sensitive to sample ordering, therefore, giving unstable and suboptimal SNP ranking in different runs when sample order is permuted. However, we found that such an unstable behavior can be effectively utilized in an ensemble learning framework. Using a simple aggregation procedure based on the general theory of ensemble learning, we can vastly improve the stability and reliability of the SNP ranking generated by these algorithms.

ReliefF based algorithms are also used to perform feature selection tasks for a range of machine learning problems including gene selection in microarray analysis. This implies our findings are not limited to the field of gene-gene interaction identification in GWA studies, and may have relevance to the broader machine learning community. Although we recognize that the sample order sensitivity problem is of less relevant to continuous datasets since tie-breaking is less likely to occur, the potential problem caused by tie-breaking in a *k*NN procedure is still noteworthy in the development of new algorithms.

Our work indicates that new algorithms should be validated against a range of criteria. Many bioinformatics algorithms have been developed to perform such filtering task. These algorithms are mostly assessed and compared based on its objective, in our situation, how well can a filtering algorithm retain functional SNP pairs. However, much less focus has been placed on analyzing whether the results generated by a SNP filtering algorithm satisfy a set of desirable properties. The sample order dependency property in this paper is one such example as it is not natural to expect the SNP ranking to change due to reordering the samples in a dataset. In fact, the importance of validating a bioinformatics algorithm and its software implementation is increasingly being recognized [29], and we believe that systematically validating an algorithm against a range of desirable property of its behavior is becoming increasingly important as biological interpretation are increasingly drawn from results produced by bioinformatics programs.

## Conclusion

We proposed an ensemble approach for gene-gene interaction filtering of GWA study dataset. Our approach aggregates the ranking scores of each SNP generated from multiple runs of RelieF or TuRF with sample-order permuted datasets. Such an ensemble method is robust to sample order dependency observed in commonly used ReliefF and TuRF algorithm. Based on the analysis using a number of real and simulated datasets, we demonstrated that the proposed approach can produce much more stable SNP ranking. In addition, the ensemble of TuRF performed the best in retaining interaction SNP pairs, superseding the performance of other traditional methods as well as state-of-the-art ReliefF-based algorithms.

## Availability

The software of ReliefF-E and TuRF-E are available from:

http://www.cs.usyd.edu.au/~yangpy/software/EnsembleFilter.html

## Competing interests

The authors declare that they have no competing interests.
